# Vulvar Extramammary Paget Disease: Diagnostic Challenge and Surgical Management in a Case Report

**DOI:** 10.3390/healthcare14142096

**Published:** 2026-07-14

**Authors:** Farah Karam, Zakhia El Beaino, Charles Sakr, Georges Nammour, Amjad Kanaan

**Affiliations:** 1 Faculty of Medicine and Medical Sciences, University of Balamand, Kalhat, Tripoli P.O. Box 100, Lebanon; farahwkaram@gmail.com (F.K.); charlessakr@yahoo.com (C.S.); 2 Pathology Department, Foch Hospital, 92150 Suresnes, France; zakelbeainou@gmail.com; 3 Pôle Mère-Enfant: Robert G. Sacy Obstetrics and Gynecology Department, Beirut Governmental Hospital Karantina, Beirut, Lebanon; 4 School of Medicine and Medical Sciences, Holy Spirit University of Kaslik, Jounieh P.O. Box 446, Lebanon; georgesnammour001@gmail.com

**Keywords:** vulvar extramammary Paget disease, EMPD, vulvar neoplasm, vulvectomy, case report

## Abstract

**Background:** Vulvar extramammary Paget disease (EMPD) is a rare intraepithelial adenocarcinoma that typically affects postmenopausal women and often mimics benign inflammatory or infectious vulvar conditions, leading to delayed diagnosis. Optimal diagnosis and therapy remain challenging due to its nonspecific presentation and variable extent of disease. **Case Presentation:** We report a 58-year-old Lebanese postmenopausal woman with more than one year of persistent vulvar pruritus and an erythematous lesion refractory to topical antifungal and corticosteroid therapy. Clinical evaluation revealed a 5 cm vulvar lesion without lymphadenopathy. Vulvar biopsy confirmed extramammary Paget disease, and staging work-up excluded associated malignancies. Although histology suggested non-invasive disease, the lesion was extensive with ill-defined margins and was reviewed by a multidisciplinary tumor board. A key feature of this case is the discordance between non-invasive biopsy findings and the decision to proceed with radical surgery due to concern for occult invasion. The patient underwent radical vulvectomy with left superficial and deep inguinal lymph node dissection. Final histopathology confirmed disease confined to the epidermis with negative margins and no nodal involvement (0/8). Postoperative imaging at 6 months showed no recurrence or metastasis. **Conclusions:** This case highlights prolonged diagnostic delay despite multiple treatments and the management challenge posed by extensive clinical disease with non-invasive biopsy findings. It underscores the importance of considering EMPD in chronic refractory vulvar lesions. Management should be individualized in multidisciplinary settings. Given the short follow-up, long-term outcomes cannot be established, and careful surveillance remains essential due to the risk of late recurrence.

## 1. Background

Extramammary Paget disease (EMPD) is a rare intraepithelial adenocarcinoma that most commonly arises in apocrine gland-bearing skin, with the vulva representing the most frequent site of involvement.

Histopathological examination remains the diagnostic gold standard, demonstrating characteristic Paget cells within the epidermis, supported by immunohistochemical staining. Although most cases are non-invasive, careful evaluation is required to exclude dermal invasion and associated or underlying malignancies. Surgical excision remains the mainstay of treatment. However, recurrence is common, necessitating long-term follow-up.

In this report, we describe a 58-year-old Lebanese postmenopausal woman with vulvar EMPD whose diagnosis was delayed despite multiple medical consultations and empiric treatments for presumed benign vulvar conditions. The case highlights the diagnostic challenges of this rare malignancy and the importance of early biopsy in persistent vulvar lesions. It also emphasizes the role of multidisciplinary evaluation in guiding the management of extensive disease with discordant clinical and histopathological findings.

## 2. Case Presentation

A 58-year-old Lebanese postmenopausal woman (menopause at age 52), gravida 4 para 4, with a medical history of hypertension, type 2 diabetes mellitus, hypothyroidism following thyroidectomy, and excess weight (BMI 28 kg/m^2^), presented with persistent vulvar pruritus and a progressively enlarging erythematous vulvar lesion. She was a chronic smoker with a 40 pack-year history. There was no personal or family history of malignancy.

The patient had reported symptoms for more than one year prior to presentation and had consulted multiple physicians during this period. She received repeated treatments, including topical and systemic antifungal agents as well as topical corticosteroids, with no clinical improvement. The prolonged symptoms significantly affected her quality of life, with persistent discomfort and sleep disturbance.

Clinical examination revealed a 5 cm erythematous vulvar lesion without palpable inguinal lymphadenopathy. Speculum examination was unremarkable.

Given the chronicity and nonspecific appearance of the lesion, the initial differential diagnosis included chronic vulvar dermatitis, vulvovaginal candidiasis, lichen sclerosus, psoriasis, Bowen disease, and melanoma. These conditions were considered based on overlapping clinical features such as pruritus, erythema, and chronic eczematous changes. However, the lack of response to appropriate empirical therapy raised suspicion for an underlying neoplastic process.

**Figure 1:**
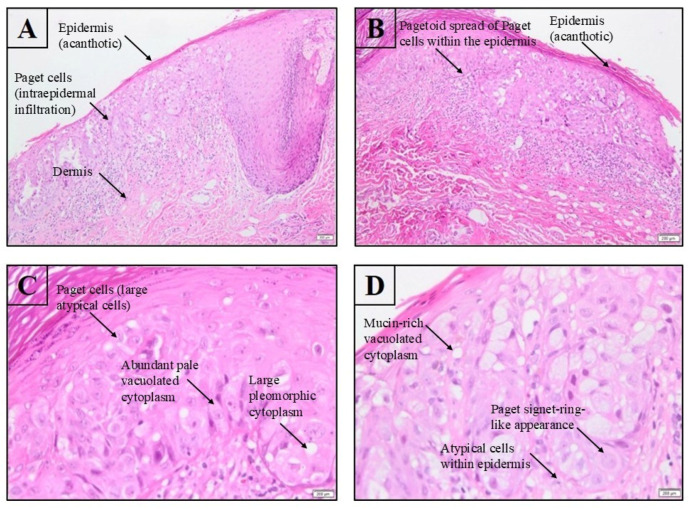
Histopathology of the initial vulvar biopsy. (**A**–**D**). Histopathological features of vulvar extramammary Paget disease (EMPD) on punch biopsy of our patient. The epidermis shows infiltration by large atypical intraepidermal cells (Paget cells) with abundant pale to vacuolated cytoplasm and pleomorphic hyperchromatic nuclei, present singly and in clusters within an acanthotic stratified squamous epithelium. Focal epithelial erosion is present with a mild chronic inflammatory infiltrate in the underlying dermis. (**A**,**B**) Extramammary Paget disease with large pale Paget cells scattered within the epidermis (H&E, ×4). (**C**,**D**) Pagetoid pale cells showing vacuolated cytoplasm, pleomorphic nuclei, and prominent nucleoli within the epidermis (H&E, ×40); the scale represents 200 μm.

Due to persistent symptoms and lack of response to treatment, a vulvar punch biopsy was performed. Histopathological examination demonstrated an acanthotic stratified squamous epithelium infiltrated by large atypical intraepidermal cells consistent with Paget cells. These cells exhibited abundant pale eosinophilic to vacuolated cytoplasm, irregular hyperchromatic nuclei, and were present both singly and in clusters within the epidermis. Focal epithelial erosion was noted, with a mild chronic inflammatory infiltrate in the underlying dermis (Figure 1). The histological features were consistent with extramammary Paget disease (Table 1).

Histochemical staining demonstrated strong intracytoplasmic positivity for mucicarmine and periodic acid–Schiff with diastase digestion (PAS-D), supporting the diagnosis of primary EMPD.

Staging investigations were performed to exclude associated malignancies. Pelvic magnetic resonance imaging (MRI) demonstrated no uterine or ovarian abnormalities. Small bilateral external iliac lymph nodes (≤5 mm) were noted, along with a 16 mm right iliac bone lesion, which was reviewed in a multidisciplinary setting and considered benign based on stable imaging characteristics and the lack of aggressive radiologic features. Chest radiography was unremarkable. Breast imaging (mammography and ultrasound) demonstrated BI-RADS 2 findings with no suspicious lesions.

Although biopsy findings indicated non-invasive disease, the lesion was clinically extensive (approximately 5 cm) with poorly defined margins, raising concern for subclinical multifocal spread. In addition, the known limitations of punch biopsy in fully excluding invasive foci were considered, particularly sampling error in heterogeneous lesions.

Given these findings, the case was reviewed by a multidisciplinary tumor board including gynecology, pathology, radiology, and oncology specialists. Following consensus, definitive surgical management was recommended.

**Figure 2:**
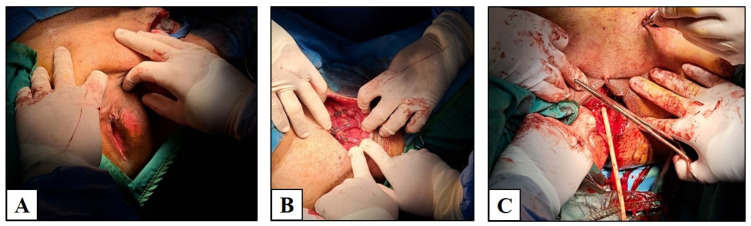
Surgical Approach for Invasive Vulvar EMPD: Case Illustration. (**A**–**C**). Intraoperative sequence illustrating the management of invasive vulvar Paget disease in our patient. (**A**) Pre-incision evaluation of the erythematous vulvar lesion prior to excision. (**B**) Inguinal dissection showing resection of deep lymph nodes as part of surgical staging. (**C**) Advanced exposure of the vulvar and inguinal regions during radical vulvectomy with lymphadenectomy.

The patient underwent radical vulvectomy with left superficial and deep inguinal lymph node dissection performed by an experienced gynecologic surgical team. The postoperative course was uneventful, and the patient was discharged on postoperative day 3 in stable condition (Figure 2).

Gross examination of the surgical specimen revealed no visible lesion. Histopathological analysis confirmed residual vulvar extramammary Paget disease confined to the epidermis without dermal invasion. Surgical margins were negative for disease. All eight excised inguinal lymph nodes were free of metastatic involvement (0/8).

Immunohistochemistry demonstrated tumor cells positive for cytokeratin 7 (CK7) and negative for cytokeratin 20 (CK20), S100, and HMB-45, supporting a diagnosis of primary EMPD and excluding melanoma and secondary gastrointestinal or urothelial origin (Table 2).

Postoperatively, the patient was managed with zinc oxide and scheduled for clinical follow-up every three months with vulvar examination and pelvic assessment, along with surveillance pelvic MRI every six months.

A follow-up pelvic MRI performed at six months demonstrated stable postoperative changes with no evidence of recurrence or metastatic disease.

At the last follow-up, the patient remained clinically stable with no signs of disease recurrence (Table 3).

## 3. Discussion

Extramammary Paget disease is a rare intraepithelial adenocarcinoma arising predominantly in apocrine gland-bearing skin and accounting for less than 1% of vulvar malignancies [11]. It most commonly affects postmenopausal women between the fifth and eighth decades of life and typically presents with nonspecific symptoms including persistent pruritus, erythema, scaling, burning, or eczematous lesions [11,12,13]. Owing to its clinical resemblance to benign dermatologic conditions such as dermatitis, psoriasis, lichen sclerosus, and fungal infections, EMPD is frequently misdiagnosed, resulting in delayed diagnosis and treatment [12,14,15]. Consequently, biopsy remains essential in patients with persistent vulvar symptoms that fail to respond to conventional therapy.

EMPD is classified as either primary or secondary disease. Primary EMPD originates within the epidermis and may be non-invasive or invasive, whereas secondary EMPD represents epidermotropic spread from an underlying malignancy, most commonly of gastrointestinal or urothelial origin [16,17,18]. Histopathological examination remains the diagnostic gold standard and demonstrates characteristic Paget cells, which are large atypical epithelial cells with abundant pale or vacuolated cytoplasm and prominent nuclei [4]. Histochemical stains such as PAS-D and mucicarmine typically demonstrate intracytoplasmic mucopolysaccharides and support the diagnosis [6]. Immunohistochemistry further assists in distinguishing primary EMPD from secondary disease and other diagnostic mimickers. Primary EMPD characteristically demonstrates positivity for CK7 and negativity for CK20 and S100, while additional markers including GCDFP-15, CDX-2, and uroplakin II/III may aid in determining the site of origin [6,7,9].

Because EMPD may be associated with synchronous or underlying malignancies in approximately 12–33% of cases, comprehensive evaluation for internal malignancy is recommended and should be tailored according to patient age, symptoms, and lesion location [19]. Appropriate assessment may include investigation of the breast, gastrointestinal tract, and urinary tract to exclude secondary disease or associated malignancies. In the present case, breast imaging and pelvic MRI were performed, and no clinical or radiological suspicion of gastrointestinal or urinary tract involvement was identified. Therefore, further endoscopic evaluation was not performed and was deemed unnecessary by the multidisciplinary team in the absence of suggestive symptoms.

Surgical excision remains the cornerstone of treatment for localized EMPD [20]. However, management is often challenging because the disease frequently extends microscopically beyond clinically visible margins in a multifocal and irregular pattern [21,22]. This subclinical spread contributes to the high recurrence rates reported after surgery, even when resection margins are histologically negative [23,24]. Recurrence rates ranging from 16% to 44% have been reported in surgically treated patients, emphasizing the need for prolonged surveillance [25]. Although surgery remains the standard treatment for localized lesions, alternative therapeutic approaches, including topical imiquimod, radiotherapy, photodynamic therapy, and systemic therapies, may be considered in selected patients with recurrent, extensive, or unresectable disease [24,26,27].

The prognosis of EMPD is largely determined by the depth of invasion and lymph node status. Patients with non-invasive or microinvasive disease generally demonstrate excellent long-term outcomes, whereas dermal invasion, lymphovascular involvement, and nodal metastasis are associated with significantly poorer survival [16,28]. Consequently, accurate pathological assessment remains critical for prognostication and treatment planning.

The present case reflects several of the diagnostic and therapeutic challenges reported in the literature. The patient experienced persistent vulvar symptoms for more than one year and underwent multiple consultations and empiric treatments with antifungal agents and corticosteroids before a biopsy was performed. This clinical course is consistent with the well-recognized tendency of EMPD to mimic benign vulvar dermatoses, resulting in delayed diagnosis [21].

Diagnosis was established by biopsy demonstrating characteristic Paget cells confined to the epidermis, with PAS-D and mucicarmine positivity and a CK7-positive/CK20-negative immunophenotype. These findings supported the diagnosis of primary EMPD and excluded melanoma and secondary gastrointestinal or urothelial malignancies [7,8]. Comprehensive staging investigations, including pelvic MRI and breast imaging, revealed no evidence of associated malignancy, supporting the diagnosis of primary vulvar EMPD. The identified iliac bone lesion was reviewed by multiple radiologists and remained consistent with a benign finding. The lesion demonstrated stable imaging characteristics without cortical destruction, a soft tissue component, or metabolic suspicion, supporting a non-aggressive etiology.

Although biopsy suggested non-invasive disease, management required multidisciplinary evaluation because of the lesion’s clinical extent, poorly defined margins, and the recognized limitations of partial biopsy sampling in completely excluding focal invasion [7]. In particular, punch biopsy may underestimate disease depth and multifocal microscopic spread, especially in heterogeneous lesions of large size, as seen in this patient. The case was therefore reviewed by a multidisciplinary tumor board involving gynecology, pathology, radiology, and oncology specialists. Following a consensus discussion, definitive surgical management was recommended.

The decision to proceed with radical vulvectomy and inguinal lymph node dissection despite non-invasive biopsy findings was based on several factors: the large lesion size (approximately 5 cm), ill-defined clinical margins, and concern for occult dermal invasion not captured by limited biopsy sampling. Although lymph node dissection is not routinely indicated in non-invasive EMPD, it may be considered in selected high-risk cases where invasion cannot be confidently excluded. This approach was therefore precautionary and MDT-driven rather than based on confirmed nodal disease.

Radical vulvectomy with inguinal lymph node dissection was performed and demonstrated residual EMPD confined to the epidermis, negative surgical margins, and the absence of nodal metastasis.

At the six-month follow-up, the patient remained clinically stable without evidence of local recurrence or metastatic disease. The absence of dermal invasion and nodal involvement suggests a favorable prognosis. Nevertheless, given the recognized propensity of EMPD for late local recurrence, continued long-term surveillance remains warranted.

## 4. Conclusions

Vulvar extramammary Paget is an uncommon malignancy that often presents with nonspecific symptoms and may mimic benign dermatologic conditions, leading to delayed diagnosis. This case highlights the importance of maintaining a high index of suspicion and performing early biopsy in persistent vulvar lesions. Histopathological evaluation remains the cornerstone of diagnosis, while surgical excision continues to represent the mainstay of treatment. Given the risk of recurrence, long-term clinical surveillance is essential even in non-invasive disease. Early recognition and multidisciplinary management are key to optimizing outcomes in patients with vulvar EMPD.

This case should be interpreted within the limitations of a single-patient report and a relatively short follow-up period of 6 months, which does not allow assessment of long-term recurrence risk. Emerging research suggests that targeted therapies and immunotherapy may play a role in advanced or refractory disease in the future; however, their clinical utility remains investigational.

## Figures and Tables

**Table 1:** Histological and cytological features of EMPD. Histological and cytological features of EMPD and related entities. Toker cells must be distinguished from Paget cells, particularly using immunohistochemical staining. Invasive disease is identified by dermal infiltration by Paget cells.

Feature	Description	Reference(s)
Toker cells	Benign clear epithelial cells, CK7 positive, without atypia	[1,2,3]
Paget cells	Large atypical cells with pale vacuolated cytoplasm and pleomorphic nuclei	[4,5]
Special stains (PAS-D, mucicarmine)	Highlight intracytoplasmic mucopolysaccharides in Paget cells	[6]
Invasive disease criteria	Paget cells breach basement membrane and infiltrate dermis	[7]

**Table 2:** Immunohistochemical profile of EMPD. Immunohistochemical markers commonly used in the diagnosis of EMPD. CK7 and CAM5.2 positivity support a diagnosis of EMPD, while negativity for CK20 and S100 helps distinguish it from other neoplasms such as melanoma or colorectal carcinoma. Additional markers aid in differentiating primary from secondary disease.

Marker	Expression in EMPD	Diagnostic Significance	Reference(s)
CK7	Positive	Supports EMPD; helps distinguish from secondary gastrointestinal/urothelial disease	[5,8]
CAM5.2	Positive	Confirms epithelial origin	[8]
GCDFP-15	Variable positive (~30–52%)	Supports primary cutaneous origin	[5,9]
CK20	Negative	Helps exclude colorectal and urothelial origin	[9,10]
S100	Negative	Excludes melanoma	[8,10]
CDX-2	Positive in secondary EMPD	Suggests gastrointestinal origin when present	[9]
Uroplakin II/III	Positive in secondary EMPD	Suggests urothelial origin when present	[9]

**Table 3:** **CARE Timeline of a Patient with Vulvar Extramammary Paget Disease.** Chronological timeline summarizing the clinical presentation, diagnostic evaluation, multidisciplinary decision-making, surgical management, histopathological findings, and follow-up course of a 58-year-old patient with vulvar extramammary Paget disease.

Time Point	Clinical Event
>12 months before presentation	Onset of vulvar pruritus and erythematous lesion
Months 0–12	Multiple consultations in primary care and specialty clinics; empiric treatments with topical antifungals and corticosteroids without improvement
At presentation (Month 0)	Clinical evaluation: 5 cm vulvar erythematous lesion, no inguinal lymphadenopathy
Shortly after presentation	Vulvar punch biopsy performed due to persistent symptoms and suspicious chronic lesion
Pathology (shortly after biopsy)	Diagnosis of extramammary Paget disease (CK7+, CK20−, S100−, PAS-D+, mucicarmine+)
Staging work-up	Pelvic MRI, chest radiography, breast imaging (mammography and ultrasound); iliac bone lesion reviewed and considered benign
MDT discussion	Multidisciplinary tumor board review (gynecology, pathology, radiology, oncology)
Surgical management	Radical vulvectomy with left superficial and deep inguinal lymph node dissection
Postoperative pathology	EMPD confined to the epidermis, negative margins, 0/8 lymph nodes positive
3 months post-op	Clinical follow-up: no recurrence
6 months post-op	MRI: no recurrence or metastasis
Last follow-up	Patient clinically stable, no evidence of disease recurrence

## Data Availability

Data available on request due to restrictions. As this manuscript is a case report involving patient data, certain identifying information, including the patient’s name and hospital medical record details, cannot be publicly shared in order to maintain patient confidentiality and privacy.

## Abbreviations

Body Mass Index (BMI); Cytokeratin 7 (CK7); Cytokeratin 20 (CK20); Extramammary Paget Disease (EMPD); Estrogen Receptor (ER); Gross Cystic Disease Fluid Protein-15 (GCDFP-15); Human Melanoma Black-45 (HMB-45); Immunohistochemistry (IHC); Multidisciplinary Tumor Board (MDT); Magnetic Resonance Imaging (MRI); Periodic Acid–Schiff with Diastase digestion (PAS-D); S100 Protein (S100); Squamous Cell Carcinoma (SCC); Sentinel Lymph Node (SLN); Sentinel Lymph Node Biopsy (SLNB).

## References

[1] Smith A.A. (2019). Pre-Paget cells: Evidence of keratinocyte origin of extramammary Paget’s disease. Intractable Rare Dis. Res..

[2] Nofech-Mozes S., Hanna W. (2009). Toker cells revisited. Breast J..

[3] Willman J.H., Golitz L.E., Fitzpatrick J.E. (2005). Vulvar clear cells of Toker: Precursors of extramammary Paget’s disease. Am. J. Dermatopathol..

[4] Delport E.S. (2013). Extramammary Paget’s disease of the vulva: An annotated review of the current literature. Australas. J. Dermatol..

[5] Tsunemi Y., Saeki H., Kikuchi K., Tamaki K., Sato S. (2009). Extramammary Paget’s disease with intracytoplasmic lumen formation. J. Dermatol..

[6] Nabavizadeh R., Vashi K.B., Nabavizadeh B., Narayan V.M., Master V.A. (2022). Extramammary Paget’s disease: Updates in the workup and management. Asian J. Urol..

[7] Kibbi N., Owen J.L., Worley B., Wang J.X., Harikumar V., Downing M.B., Aasi S.Z., Aung P.P., Barker C.A., Bolotin D. (2022). Evidence-Based Clinical Practice Guidelines for Extramammary Paget Disease. JAMA Oncol..

[8] Chung J., Kim J.Y., Gye J., Namkoong S., Hong S.P., Park B.C., Kim M.H. (2013). Extramammary Paget’s Disease of External Genitalia with Bowenoid Features. Ann. Dermatol..

[9] Ohnishi T., Watanabe S. (2000). The use of cytokeratins 7 and 20 in the diagnosis of primary and secondary extramammary Paget’s disease. Br. J. Dermatol..

[10] Jang E.J., Bae Y.K., Shin D.H., Lee D.J. (2016). Extramammary Paget’s Disease Combined with Squamous Cell Carcinoma In Situ of the Vulva: A Case Report and Differential Diagnosis. Ann. Dermatol..

[11] Simonds R.M., Segal R.J., Sharma A. (2019). Extramammary Paget’s disease: A review of the literature. Int. J. Dermatol..

[12] Bajracharya A., Shrestha S., Singh M., Shrestha S., Lama S., Singh J. (2022). Vulvar Paget’s disease associated with squamous cell carcinoma: A case report. Ann. Med. Surg..

[13] Morris C.R., Hurst E.A. (2020). Extramammary Paget Disease: A Review of the Literature-Part I: History, Epidemiology, Pathogenesis, Presentation, Histopathology, and Diagnostic Work-up. Dermatol. Surg..

[14] Konstantinova A.M., Kazakov D.V. (2021). Extramammary Paget disease of the vulva. Semin. Diagn. Pathol..

[15] Tomar T.S., Sambasivan S., Nair R.P., Thomas S., Ramadas P.T. (2016). Extra Mammary Paget’s Disease of Vulva-a Case Report. Indian J. Surg. Oncol..

[16] Ishizuki S., Nakamura Y. (2021). Extramammary Paget’s Disease: Diagnosis, Pathogenesis, and Treatment with Focus on Recent Developments. Curr. Oncol..

[17] Scarbrough C.A., Vrable A., Carr D.R. (2019). Definition, Association with Malignancy, Biologic Behavior, and Treatment of Ectopic Extramammary Paget’s Disease: A Review of the Literature. J. Clin. Aesthetic Dermatol..

[18] Sawada Y., Bito T., Kabashima R., Yoshiki R., Hino R., Nakamura M., Masanori S., Tokura Y. (2010). Ectopic extramammary Paget’s disease: Case report and literature review. Acta Derm. Venereol..

[19] Karam A., Dorigo O. (2012). Treatment outcomes in a large cohort of patients with invasive Extramammary Paget’s disease. Gynecol. Oncol..

[20] Anjana J.S., Rema P., Suchetha S., Ranjith J.S., Rao A.B., Preethi T.R. (2021). Impact of Surgery on Extramammary Paget’s Disease Vulva: A Case Series. Indian J. Surg. Oncol..

[21] Parashurama R., Nama V., Hutson R. (2017). Paget’s Disease of the Vulva: A Review of 20 Years’ Experience. Int. J. Gynecol. Cancer Off. J. Int. Gynecol. Cancer Soc..

[22] Roh H.J., Kim D.Y., Kim J.H., Kim Y.M., Kim Y.T., Nam J.H. (2010). Paget’s disease of the vulva: Evaluation of recurrence relative to symptom duration, volumetric excision of lesion, and surgical margin status. Acta Obs. Gynecol. Scand..

[23] Cho A., Kim D.-Y., Suh D.-S., Kim J.-H., Kim Y.-M., Kim Y.-T., Park J.-Y. (2023). Outcomes and prognostic factors of surgically treated extramammary Paget’s disease of the vulva. J. Gynecol. Oncol..

[24] Edey K.A., Allan E., Murdoch J.B., Cooper S., Bryant A. (2019). Interventions for the treatment of Paget’s disease of the vulva. Cochrane Database Syst. Rev..

[25] Bae J.M., Choi Y.Y., Kim H., Oh B.H., Roh M.R., Nam K., Chung K.Y. (2013). Mohs micrographic surgery for extramammary Paget disease: A pooled analysis of individual patient data. J. Am. Acad. Dermatol..

[26] Borella F., Preti M., Vieira-Baptista P., Pérez-López F.R., Bertero L., Gallio N., Micheletti L., Benedetto C. (2022). Vulvar Paget’s disease: Outcomes of 51 patients treated with imiquimod cream. Maturitas.

[27] Nakamura Y., Mizukami H., Tanese K., Fusumae T., Hirai I., Amagai M., Takamatsu R., Nakamura K., Nishihara H., Takimoto T. (2023). Role of androgen signaling in androgen receptor-positive extramammary Paget’s disease: Establishment of organoids and their biological analysis as a novel therapeutic target. J. Dermatol. Sci..

[28] Caruso G., Barcellini A., Mazzeo R., Gallo R., Vitale M.G., Passarelli A., Mangili G., Pignata S., Palaia I. (2023). Vulvar Paget’s Disease: A Systematic Review of the MITO Rare Cancer Group. Cancers.

